# Variations in Emergency Service Utilization among Cancer Survivors: Results from the Pan-Canadian Experiences of Cancer Patients in Transition Study Survey

**DOI:** 10.1155/2023/5056408

**Published:** 2023-03-16

**Authors:** Megan Delisle, Ying Wang, Margaret I. Fitch, Kalki Nagaratnam, Amirrtha Srikanthan

**Affiliations:** ^1^Division of General Surgery, The Ottawa Hospital, Ottawa, Ontario, Canada; ^2^University of Ottawa, Ottawa, Ontario, Canada; ^3^University of British Columbia, Vancouver, British Columbia, Canada; ^4^BC Cancer Vancouver, Vancouver, British Columbia, Canada; ^5^Bloomberg Faculty of Nursing, University of Toronto, Toronto, Ontario, Canada; ^6^Department of Medical Oncology, The Ottawa Hospital Cancer Center, Ottawa, Ontario, Canada

## Abstract

**Purpose:**

The objective of this study was to examine variations in emergency service utilization (ESU) among cancer survivors during the first year after completing primary cancer treatment.

**Methods:**

In 2016, the Canadian Partnership Against Cancer collected survey responses from cancer survivors across Canada about self-reported ESU after completing primary cancer treatment. We included survey respondents diagnosed with nonmetastatic breast, hematologic, colorectal, melanoma, or prostate cancer. Multivariable, multinomial logistic regression analysis was used to examine factors associated with cancer survivors' ESU.

**Results:**

Of the 5,774 cancer survivors included in our analysis, 22% reported ESU during the first year after completing their primary cancer treatment, 16% reported ESU one to three times, and 6% reported ESU more than three times. Factors significantly associated with frequent ESU included younger age, colorectal and hematologic cancers, more frequent primary care provider and oncology specialist visits, single or retired status, lower income, and self-reported lower quality of life.

**Conclusion:**

Our study identified factors associated with more frequent ESU among cancer survivors in the first year after completing primary cancer treatment. These factors highlight differences in cancer survivors' demographics, their ability to access and need for healthcare services, and the complexity of using ESU as a metric for quality improvement in survivorship care. These variations must be considered in quality improvement initiatives.

## 1. Background

In countries such as Canada, the cancer incidence is estimated to increase by 32% between 2020 and 2040, with more than 60% of adults now living five years or more [1–4]. As a result, there are more cancer survivors than ever before. The growing number of cancer survivors results in greater demand for healthcare that meets their ongoing, specialized needs [1, 4].

Cancer survivors have unique healthcare needs due to multiple factors. These needs vary depending on individual risk of cancer recurrence, development of new and secondary cancers, ongoing physical and psychosocial effects of treatment, and noncancer-related comorbidities [5]. Research has shown that healthcare for cancer survivors frequently does not meet their needs, owing partly to a lack of coordination, insufficient resources, workforce shortages, and rising healthcare expenses [5, 6]. Improving the quality of survivorship care is a priority [6, 7].

Quality metrics have been proposed to measure, evaluate, and improve the quality of survivorship care. Healthcare utilization is one such metric [5, 8]. Healthcare utilization represents the interrelated complexities of the availability and accessibility of needed healthcare services by patients [9]. Ideally, optimized healthcare utilization would result in improved health, quality of life, and patient experience through efficient use of resources. Certain measures of healthcare utilization can reveal areas of fragmentation and preventable inefficiency. In this regard, emergency service utilization (ESU) is a healthcare utilization metric proposed to measure the quality of survivorship care, as it has been associated with high resource utilization that does not always result in improved outcomes [10]. However, studies assessing ESU in cancer survivors and strategies to improve it are lacking.

In 2016, the Canadian Partnership Against Cancer (CPAC) developed and distributed the Experiences of Cancer Patients inTransition Study survey, herein referred to as the Transition Study, to over 40,000 randomly selected cancer survivors to understand experiences with follow-up cancer care one to three years after completing primary treatment. The primary objective of this study was to understand the factors associated with cancer survivors' self-reported ESU during the first year after completing primary cancer treatment. The secondary objective was to assess ESU, primary care provider (PCP), and oncology specialist utilization three years after completing primary cancer treatment.

## 2. Methods

This article was reported in accordance with the Checklist for Reporting of Survey Studies (CROSS) [11].

### 2.1. Survey Development

Fitch et al. previously described the Transition Study survey development and dissemination in detail [12]. In summary, CPAC worked with all ten provincial cancer agencies across Canada to develop the survey guided by stakeholders. The survey was piloted with cancer survivors. The final Transition Study survey included closed- and open-ended questions, was available in both official languages of Canada (French and English), took 30 to 45 minutes to complete, and was available on paper or online. A copy of the full survey is publicly available on the CPAC system performance website [13].

### 2.2. Survey Dissemination

CPAC collaborated with the ten provincial cancer agencies across Canada to disseminate the survey. The eligibility criteria included adult survivors over 30 years of age with one of the following nonmetastatic cancer types: breast, prostate, colorectal, and melanoma, or selected metastatic hematological cancers (i.e., Hodgkin's lymphoma, diffuse B-cell lymphoma, acute myelogenous leukemia, and acute lymphocytic lymphocyte leukemia). Adolescent and young adult (AYA) survivors between 18 and 29 years old with any nonmetastatic cancer types or metastatic testicular cancer were also eligible. Survivors were defined in the Transition Study as those who completed primary cancer treatment.

Each province obtained ethics and privacy approval before data collection. Informed consent was obtained from respondents prior to engaging with the survey. Between June and October 2016, 40,790 survey packages were mailed out. The number of surveys for distribution was calculated for the adult sample such that 95% confidence intervals would have a width of no more than ±5% by disease sites and provinces for a percentage assumed to be 50% and an assumed response rate of 30%. Estimates of the number of eligible survivors per province were based on national disease site prevalence and incidence by province. As smaller provinces were unlikely to have enough survivors within disease sites to achieve the desired confidence interval precision, all eligible survivors were contacted. A random sample within the cancer disease site was chosen for larger provinces and disease sites where the number of eligible survivors was anticipated to be greater than the required number. The final sample was sufficient to achieve precision of at least ±3% by disease site for all provinces combined.

### 2.3. Study Population

The subset of respondents we included was diagnosed with nonmetastatic breast, hematologic, colorectal, melanoma, or prostate cancer. We excluded respondents who did not complete the survey themselves, or whose age, sex, or year of diagnosis were unknown, or whose last treatment date was unknown or more than five years ago. We also excluded respondents for whom the primary outcome was unavailable (i.e., those who, when asked how many times they used emergency services in the first year after cancer treatment, responded that they did not know and could not remember or those services did not apply to them).

### 2.4. Outcome

In a previous study, we examined the influence of individual and structural factors on patients' experience with survivorship care among respondents who completed the Transition Study [14]. The primary outcome of this study was cancer survivors' self-reported ESU in the first year after completing cancer treatment, defined using responses to one of the subquestions from question 23 of the Transition Study survey (Supplementary Material Table 1). This subquestion asked respondents how often they visited an emergency room or urgent care center (herein referred to as emergency services) in the first year after primary cancer treatment.

The secondary outcome was emergency service, primary care provider (PCP), and oncology specialist utilization within three years after completing primary cancer treatment. This information was obtained from other subquestions from question 23 (Supplementary Material Table 1). PCP was defined as a family doctor, general practitioner, or nurse practitioner. An oncology specialist was defined in the survey as an oncologist, hematologist, surgeon, or other cancer specialist.

### 2.5. Factors Associated with ESU during the First Year after Completing Primary Cancer Treatment

Using expert knowledge, existing evidence, and the Andersen Model of health service use, we hypothesized factors collected in the Transition Study survey that may be associated with cancer survivors' ESU [9, 15–17]. Survey questions and responses corresponding to these factors are included in Supplementary Material Table 1. In addition to these factors, we created a variable assessing unmet needs among cancer survivors, a binary variable derived from methods previously used in the publication of the Transition Study results described by Shakeel et al. (Supplementary Material) [18].

### 2.6. Statistical Analysis

The differences between respondents included in and those excluded from our analysis were compared using bivariate analyses. Factors associated with survivors' ESU in the first year following cancer treatment were assessed using bivariate analyses and multivariable, multinomial logistic regression analysis. The reference category for the multinomial regression analysis was no ESU in the first year after cancer treatment. This category was compared to two others, namely, ESU one to three times and more than three times.

Factor levels within categorical variables were combined when necessary to ensure an adequate sample size per category. Missing data were included as factor levels in categorical variables where appropriate. A two-sided*p* value <0.05 was considered statistically significant for the multivariable analysis. All analyses were performed with StataMP, version 17.0 (StataCorp LLC).

## 3. Results

A total of 13,319 respondents returned the survey (response rate 33%). Based on our defined inclusion criteria, 5,774 unique respondents were part of this analysis. We excluded the following respondents: those who did not have nonmetastatic breast, hematologic, colorectal, melanoma, or prostate cancer (*n* = 3,904), those who had primary outcomes missing (*n* = 3,170), those who did not complete the survey themselves (*n* = 237), whose age or sex was unknown (*n* = 34), whose year of diagnosis was unknown (*n* = 35), or whose last treatment date was unknown or more than five years ago (*n* = 165).

### 3.1. Comparison of Included versus Excluded Study Population Personal and Clinical Characteristics

Compared to excluded respondents, included respondents were more likely to be between 45 and 64 years old (41% included vs. 27% excluded), have private insurance (51% included vs. 38% excluded), work full- or part-time (34% included vs. 23% excluded), earn over $75,000 annually (31% included vs. 19% excluded), and have an unmet emotional (69% included vs. 58% excluded) or physical (66% included vs. 55% excluded) need. Included patients were also more likely to have breast cancer (35% included vs. 22% excluded), have undergone cancer treatment that included surgery (72% included vs. 61% excluded), radiotherapy (49% included vs. 39% excluded), or systemic therapy (80% included vs. 70% excluded), and have completed their last cancer treatment between one and three years ago (54% included vs. 40% excluded) (Table 1).

### 3.2. Personal and Clinical Characteristics of the Included Study Population

Most respondents in our analysis were married or partnered (76%), found it easy or very easy to ask their healthcare provider questions about their follow-up cancer care-related concerns (75%), were born in Canada (85%), spoke English most commonly at home (78%), rated their overall quality of life as good or very good (86%), rated their physical health (78%) and emotional health (78%) as good or very good, received cancer treatment that included surgery (71%) and/or systemic therapy (80%), and were not enrolled in a clinical trial (86%) (Table 1).

### 3.3. ESU in the First Year after Completing Cancer Treatment

Most respondents (78%) reported not using emergency services during the first year after cancer treatment; 16% reported using them one to three times, and 6% reported using them more than three times (Table 2).

### 3.4. Factors Associated with ESU in the Year following Cancer Treatment

We identified statistically significant factors associated with increased ESU across all levels of the multivariable, multinomial regression analysis (Tables 2 and 3). These included younger age, more frequent PCP and oncology specialist utilization, colorectal and hematologic cancers, single or retired status, speaking French most commonly at home, lower income, self-reported lower quality of life, and access to a copy of their medical records (Table 3).

Some characteristics were significantly more likely to be associated with ESU one to three times but not more than three times, such as living in a rural area, lower self-reported emotional health, receiving surgery as part of their cancer treatment, and more recent completion of cancer treatment at the time of survey completion (i.e., within the last year versus one to three years ago versus three to five years ago versus no treatment) (Table 3).

Some characteristics were significantly more likely to be associated with ESU more than three times but not one to three times, such as having an unmet practical need, more comorbidities, and clinical trial enrollment (Table 3).

### 3.5. Emergency Service, PCP, and Oncology Specialist Utilization in the Second and Third Years after Completing Primary Cancer Treatment

Compared to ESU in the first year (78%), more respondents reported not using emergency services in the second (92%) and third (94%) years after completing cancer treatment.

Emergency service, PCP, and oncology specialist utilization in the second and third years after completing cancer treatment by frequency of ESU in the first year is displayed in Table 4. Respondents who reported less ESU in the first year after cancer treatment were also less likely to report emergency service, PCP, and oncology specialist utilization in the second and third years.

## 4. Discussion

Our study identified several factors associated with more frequent ESU among cancer survivors in the first year after completing primary cancer treatment, including younger age, colorectal and hematologic cancers, more frequent PCP and oncology specialist visits, single or retired status, lower income, and self-reported lower quality of life. These variations in ESU suggest differences in cancer survivors' individual needs and ability to access healthcare services. These differences are important to consider when using ESU as a quality metric or to plan initiatives aimed at improving survivorship care.

Younger patients reported higher ESU in our study. Previous studies have shown that younger age at cancer diagnosis is associated with more side effects and disability during and after treatment [19–22]. In the Transition Study, 80 to 90% of adolescent and young adult (AYA) cancer survivors, i.e., between 18 and 29 years old, reported physical, emotional, or practical concerns and 85% reported experiencing challenges related to reduced physical capacity, pain, difficulty coping distress, and a struggle to return to normal function [19–21]. In addition, they experienced difficulty accessing nonemergent healthcare services and encountered challenges in obtaining assistance with side effects from their cancer treatment [19–22]. The higher needs in this age group, which are often less well met compared to other groups, could be one reason for increased ESU, along with differences in treatment regimens with unique side effect profiles, social functioning, and caregiver support in AYA cancer survivors. This population has been historically underrepresented in cancer care quality improvement projects, and gaps exist in our understanding of their needs and experiences [17, 23]. Further research will help inform strategies to improve the quality of survivorship care for AYA cancer survivors.

Previous publications have suggested that increased ESU is associated with limited access to routine, outpatient healthcare services [24, 25]. Interventions, such as expedited access to knowledgeable providers by phone (telehealth) or to next-day appointments, have been shown to reduce ESU [26]. Interestingly, we found that respondents with increased ESU also reported increased PCP and oncology specialist utilization. These findings suggest that other factors may be driving ESU. For example, the high usage of emergent and nonemergent healthcare services observed in our study could represent care fragmentation in a subpopulation of patients [16]. Care fragmentation occurs when individuals visit multiple healthcare providers for the same reasons and is associated with repetitive investigations, increased healthcare costs, and more frequent ESU among cancer survivors [16]. Higher emergency service, PCP, and oncology specialist utilization may also reflect the small portion of patients with the most acute care needs. These patients may benefit from more intensive longitudinal follow-ups [27]. Without information on the appropriateness and quality of care provided through emergency services, PCPs, and oncology specialists in the Transition Study, it is difficult to recommend ways to improve. A risk-stratified approach to cancer survivors' follow-up cancer care, where survivors with more needs have frequent contact with personalized healthcare services, may help provide higher quality survivorship care.

Another key finding in our study is that ESU differed significantly by cancer types. Patients with colorectal or hematological malignancies reported higher ESU than those with breast or prostate cancer or melanoma. In this regard, ESU is likely more clinically appropriate for individuals diagnosed with cancers with delayed treatment toxicities or more acute recurrence presentations, which are especially prevalent in the first year following treatment completion, when complications and disease relapse rates are highest [4, 28]. For example, patients with hematologic cancers may experience significant late effects of treatment, including higher needs for access to emergency services to treat recurrent infections or transfusions. Similarly, colorectal cancer patients may present with more acute symptoms related to major surgery or local relapses, such as bowel bleeding and obstruction, which require emergent workup and treatment. Future initiatives aiming to use ESU as a quality metric in survivorship care should consider this to avoid penalizing those who need access most.

Despite high variability in ESU, we see a decline in emergent and nonemergent healthcare utilization after the first year. This finding is consistent with the existing literature; long-term cancer survivors have different healthcare needs than new survivors, especially those within the first year after treatment completion when recurrence rates and toxicities are highest [29]. Our study provides insights into ESU variations within the first three years after completing cancer treatment, supporting the need to define phases of survivorship across settings for meaningful comparison of quality metrics.

Even though Canada has public, universal healthcare coverage, we found that respondents with lower income, living in rural areas, single status, and lower quality of life used emergency services more frequently. The most recent systematic review of cancer survivors' healthcare utilization published in 2012 similarly found that preventable hospitalization was higher among those with lower income, of minority race or ethnicity, and widowed or divorced [15, 30–32]. While universal healthcare reduces direct costs related to healthcare, it does not always alleviate indirect costs, such as those related to transport, childcare, and supportive care services [30–32]. In some instances, services are available to help with indirect costs, but these may not be equitably accessible. These findings highlight the need for personalized survivorship care models to ensure a healthcare system that meets everyone's needs, regardless of differing social determinants of health.

Determining whether the ESU reported in our study represents an efficient use of healthcare resources requires an understanding of the factors influencing ESU among oncology patients [33]. Research into these factors has shown that most ESU is for treatment-related side effects and complications, of which approximately 40% may be preventable [34, 35]. Still, few studies have assessed why oncology patients decide to use emergency services compared to other healthcare services. Nguyen et al. identified that oncology patients over the age of 70 in Canada who accessed emergency services during cancer treatment did so primarily because other cancer care options were unavailable or they had a life-threatening health condition [36]. Thus, some needs of cancer survivors driving ESU may be better met for certain patients through alternative healthcare services, if available. However, as seen in our study, factors contributing to ESU are varied, and the reasons behind ESU are complex.

### 4.1. Clinical Implications

Quality in healthcare is a complex and interconnected concept that can be difficult to capture, yet measuring quality in healthcare is paramount to compare and improve it. The existing definitions and frameworks can be challenging to operationalize locally driven changes [5, 37]. We demonstrate that using ESU as a quality metric alone, without a deeper understanding of contextual factors contributing to ESU, is unlikely to provide information for meaningful improvements in the quality of care [38].

Another important factor to consider when using quality metrics to guide quality improvement is how using different measures for the same concept can reflect different aspects of healthcare quality. For example, a recent review identified 14 unique ESU measures from 29 studies [39]. The authors concluded that the number or frequency of use of emergency services was believed to measure accessibility, whereas the time between arrival to the emergency department and diagnosis or treatment was believed to measure timeliness [39]. A review of cancer survivors' healthcare utilization patterns identified that it was not just the number of healthcare visits that were associated with the quality of healthcare but also the type of provider and the number of patients shared between providers [16]. In our study, we used the number of times emergency services were used over a 12-month period, which is hypothesized to reflect differential access to inpatient, acute care versus outpatient, and routine care [39]. It is important that future research should aim to understand the dimensions of healthcare quality reflected by ESU among cancer survivors across different contexts to be able to make meaningful changes with these results. Ultimately, while quality metrics are important to improve healthcare, how these metrics are defined, prioritized, and interpreted must be determined in the local context.

### 4.2. Study Limitations

Limitations of our study are the self-reported survey design and limited data generalizability. The accuracy of self-reported healthcare use varies by healthcare service types (e.g., routine healthcare, emergency services, and invasive testing), time elapsed since use, utilization frequency, and questionnaire design [40]. Memorable or highly emotional events, as is common in ESU, are more accurately recalled [40]. Despite these limitations, self-reported healthcare service utilization remains a commonly used data source in health service research. Validated self-reported healthcare use questionnaires have been developed but not yet adapted for cancer survivors, which should be done to improve the quality of future research on healthcare utilization among cancer survivors [41].

Our study findings may not be generalized to all cancer survivors in Canada because weighting was not applied due to the confidential nature of the data and the use of an external vendor for survey dissemination. Furthermore, the response rate of the Transition Study survey was 33%, and we excluded additional 57% of respondents from our analysis. In our opinion, considering the length and detail of the Transition Study survey, this response rate represents a strength compared to other surveys of this magnitude. In addition, we presented data by comparing respondents included in versus excluded from our analysis to provide transparency about to whom our findings may not apply.

We recommend caution in generalizing our findings to other healthcare systems, particularly with different insurance or survivorship care models. Factors influencing ESU are likely to differ in jurisdictions with predominately private insurance or more geographically accessible specialized oncology care. Current survivorship care is not standardized in Canada. While we controlled for some differences in survivorship care (e.g., receipt of a care plan, a copy of medical records, and providers responsible for follow-up cancer care), some aspects remain unaccounted for, and the influence of these aspects on ESU is unknown. These unaccounted aspects reflect an ongoing challenge with cancer survivorship research. Future efforts should aim to standardize data collection on survivorship care, which is of particular importance given that one of the solutions to improving the quality of survivorship care is likely to involve leveraging personalized survivorship care models.

Other limitations of our study represent future directions for research. We could not provide insights into the appropriateness and outcomes of ESU among cancer survivors in our study. Due to these limitations, it is difficult to make recommendations to improve ESU among cancer survivors as we cannot determine if variations in use represent low- or high-quality care.

## 5. Conclusion

There are an increasing number of cancer survivors who require specialized survivorship care to improve their quality of life and long-term outcomes. Our study identified several factors associated with more frequent ESU among cancer survivors in the first year after completing primary cancer treatment. These variations in ESU highlight the differences in cancer survivors' individual predisposition, ability to access, and need for survivorship care. Additional research is needed to understand if these differences represent clinically appropriate care consistent with patient preferences or result from inequities. This foundational work is necessary before ESU can be used as a quality metric in survivorship care.

## Figures and Tables

**Table 1:** Personal and clinical characteristics of cancer survivors included in and excluded from our study population.

Characteristic	Total	Excluded	Included	*p* value
	*N* = 13,319	*N* = 7,545	*N* = 5,774	
Sex				<0.001
Male	48.1 (6,411)	50.0 (3,770)	45.7 (2,641)	
Female	51.2 (6,820)	48.9 (3,687)	54.3 (3,133)	
Missing	0.7 (88)	1.2 (88)	0.0 (0)	
Age at diagnosis				<0.001
18–44	7.0 (927)	7.2 (540)	6.7 (387)	
45–64	32.7 (4,356)	26.7 (2,017)	40.5 (2,339)	
≥65	59.9 (7,975)	65.3 (4,927)	52.8 (3,048)	
Missing	0.5 (61)	0.8 (61)	0.0 (0)	
Marital status				<0.001
Single	7.2 (962)	7.7 (581)	6.6 (381)	
Married/partnered	72.8 (9,701)	70.2 (5,295)	76.3 (4,406)	
Divorced/separated/widowed	18.8 (2,500)	20.5 (1,550)	16.5 (950)	
Prefer not to answer	1.2 (156)	1.6 (119)	0.6 (37)	
Coping with challenges				<0.001
Very easy/easy	59.9 (7,976)	57.5 (4,335)	63.1 (3,641)	
Neither easy nor hard	31.6 (4,211)	32.9 (2,484)	29.9 (1,727)	
Hard/very hard	7.4 (984)	7.9 (599)	6.7 (385)	
Missing	1.1 (148)	1.7 (127)	0.4 (21)	
Sharing worries				<0.001
Very easy/easy	49.4 (6,585)	48.7 (3,678)	50.3 (2,907)	
Neither easy nor hard	29.4 (3,922)	29.7 (2,242)	29.1 (1,680)	
Hard/very hard	19.5 (2,598)	19.1 (1,438)	20.1 (1,160)	
Missing	1.6 (214)	2.5 (187)	0.5 (27)	
Asking questions				<0.001
Very easy/easy	72.7 (9,683)	71.2 (5,371)	74.7 (4,312)	
Neither easy nor hard	18.5 (2,460)	18.5 (1,399)	18.4 (1,061)	
Hard/very hard	7.2 (958)	7.6 (577)	6.6 (381)	
Missing	1.6 (218)	2.6 (198)	0.3 (20)	
Current insurance				<0.001
Government-sponsored	21.7 (2,894)	23.1 (1,744)	19.9 (1,150)	
Private	43.2 (5,750)	37.5 (2,829)	50.6 (2,921)	
No insurance	15.3 (2,039)	16.5 (1,242)	13.8 (797)	
No response/other	19.8 (2,636)	22.9 (1,730)	15.7 (906)	
Born in Canada				<0.001
Yes	82.3 (10,955)	80.3 (6,058)	84.8 (4,897)	
No	15.2 (2,022)	15.9 (1,197)	14.3 (825)	
Prefer not to answer	2.6 (342)	3.8 (290)	0.9 (52)	
Language most spoken at home				<0.001
English	72.5 (9,655)	68.5 (5,171)	77.7 (4,484)	
French	21.4 (2,846)	23.9 (1,806)	18.0 (1,040)	
Other	6.1 (818)	7.5 (568)	4.3 (250)	
Highest level of education				<0.001
≤High school diploma	36.7 (4,886)	40.7 (3,070)	31.5 (1,816)	
<University degree	34.9 (4,650)	33.0 (2,493)	37.4 (2,157)	
University degree	24.4 (3,244)	20.6 (1,558)	29.2 (1,686)	
No response	4.0 (539)	5.6 (424)	2.0 (115)	
Population density				<0.001
≤10,000 people	34.9 (4,646)	34.8 (2,627)	35.0 (2,019)	
10,000 to 50,000 people	17.4 (2,316)	18.2 (1,370)	16.4 (946)	
>50,000 people	44.6 (5,937)	42.4 (3,197)	47.5 (2,740)	
No response	3.2 (420)	4.7 (351)	1.2 (69)	
Employment				<0.001
Full- or part-time	28.2 (3,753)	23.4 (1,767)	34.4 (1,986)	
Sick leave/disability/unemployed	5.4 (714)	5.3 (398)	5.5 (316)	
Retired	59.4 (7,907)	62.3 (4,703)	55.5 (3,204)	
No response/other	7.1 (945)	9.0 (677)	4.6 (268)	
Income				<0.001
≤$25,000	12.8 (1,708)	15.0 (1,129)	10.0 (579)	
$25,000 to <$50,000	23.2 (3,090)	24.3 (1,832)	21.8 (1,258)	
$50,000 to <$75,000	16.1 (2,149)	15.3 (1,155)	17.2 (994)	
≥$75,000	24.3 (3,240)	18.9 (1,425)	31.4 (1,815)	
Prefer not to answer	23.5 (3,132)	26.6 (2,004)	19.5 (1,128)	
Overall quality of life				<0.001
Very poor/poor/fair	17.5 (2,326)	20.1 (1,517)	14.0 (809)	
Good/very good	82.0 (10,919)	79.2 (5,976)	85.6 (4,943)	
Missing	0.6 (74)	0.7 (52)	0.4 (22)	
Physical health				<0.001
Very poor/poor/fair	25.7 (3,420)	28.6 (2,159)	21.8 (1,261)	
Good/very good	73.8 (9,828)	70.5 (5,322)	78.0 (4,506)	
Missing	0.5 (71)	0.8 (64)	0.1 (7)	
Emotional health				<0.001
Very poor/poor/fair	21.2 (2,818)	22.8 (1,723)	19.0 (1,095)	
Good/very good	74.2 (9,878)	71.2 (5,375)	78.0 (4,503)	
Missing	4.7 (623)	5.9 (447)	3.0 (176)	
Unmet practical concern				<0.001
No	67.0 (8,928)	70.3 (5,305)	62.7 (3,623)	
Yes	33.0 (4,391)	29.7 (2,240)	37.3 (2,151)	
Unmet emotional concern				<0.001
No	37.0 (4,928)	41.6 (3,135)	31.1 (1,793)	
Yes	63.0 (8,391)	58.4 (4,410)	68.9 (3,981)	
Unmet physical concern				<0.001
No	40.3 (5,361)	44.9 (3,389)	34.2 (1,972)	
Yes	59.7 (7,958)	55.1 (4,156)	65.8 (3,802)	
Number of comorbidities				<0.001
None	35.4 (4,718)	32.8 (2,478)	38.8 (2,240)	
1-2	56.1 (7,468)	58.0 (4,377)	53.5 (3,091)	
≥3	8.5 (1,133)	9.1 (690)	7.7 (443)	
Cancer type				<0.001
Breast	27.5 (3,665)	21.8 (1,643)	35.0 (2,022)	
Hematologic	8.5 (1,129)	7.3 (548)	10.1 (581)	
Colorectal	18.5 (2,459)	17.2 (1,296)	20.1 (1,163)	
Melanoma	10.8 (1,445)	11.0 (827)	10.7 (618)	
Prostate	22.7 (3,018)	21.6 (1,628)	24.1 (1,390)	
Other	5.4 (723)	9.6 (723)	0.0 (0)	
Missing	6.6 (880)	11.7 (880)	0.0 (0)	
Year of cancer diagnosis				<0.001
≤2012	26.7 (3,550)	26.9 (2,029)	26.3 (1,521)	
2013	43.2 (5,749)	39.7 (2,995)	47.7 (2,754)	
≥2014	25.0 (3,325)	24.2 (1,826)	26.0 (1,499)	
Missing	5.2 (695)	9.2 (695)	0.0 (0)	
Surgery				<0.001
No	34.4 (4,579)	38.9 (2,936)	28.5 (1,643)	
Yes	65.6 (8,740)	61.1 (4,609)	71.5 (4,131)	
Radiotherapy				<0.001
No	56.8 (7,565)	61.4 (4,630)	50.8 (2,935)	
Yes	43.2 (5,754)	38.6 (2,915)	49.2 (2,839)	
Systemic therapy				<0.001
No	25.7 (3,422)	30.4 (2,291)	19.6 (1,131)	
Yes	74.3 (9,897)	69.6 (5,254)	80.4 (4,643)	
Time of last treatment				<0.001
<1 year ago	11.3 (1,507)	13.2 (995)	8.9 (512)	
1–3 years ago	46.0 (6,133)	39.7 (2,994)	54.4 (3,139)	
3–5 years ago	23.0 (3,060)	20.0 (1,508)	26.9 (1,552)	
>5 years ago	1.4 (191)	2.5 (191)	0.0 (0)	
No treatment	14.3 (1,906)	17.7 (1,335)	9.9 (571)	
Missing	3.9 (522)	6.9 (522)	0.0 (0)	
Currently receiving or received prescribed medication to prevent cancer recurrence				<0.001
No	69.6 (9,264)	69.6 (5,250)	69.5 (4,014)	
Yes	25.5 (3,400)	22.9 (1,725)	29.0 (1,675)	
Unsure/blank	4.9 (655)	7.6 (570)	1.5 (85)	
Enrolled in a clinical trial				<0.001
Yes	9.8 (1,307)	9.2 (696)	10.6 (611)	
No	83.3 (11,094)	81.4 (6,139)	85.8 (4,955)	
No response	6.9 (918)	9.4 (710)	3.6 (208)	
Times visit/speak in first 12 months—emergency room/urgent care				<0.001
None	47.7 (6,355)	24.3 (1,832)	78.3 (4,523)	
1–3 times	11.0 (1,461)	7.3 (548)	15.8 (913)	
>3 times	4.5 (604)	3.5 (266)	5.9 (338)	
Do not remember/not applicable/missing	36.8 (4,899)	64.9 (4,899)	0.0 (0)	
Times visit/speak in first 12 months—family doctor/practitioner				<0.001
Not at all	13.0 (1,726)	9.4 (707)	17.6 (1,019)	
1–3 times	36.5 (4,864)	32.6 (2,463)	41.6 (2,401)	
>3 times	34.7 (4,624)	33.8 (2,552)	35.9 (2,072)	
Do not remember/not applicable/missing	15.8 (2,105)	24.2 (1,823)	4.9 (282)	
Times visit/speak in first 12 months—oncologist/hematologist				<0.001
Not at all	6.3 (842)	4.1 (306)	9.3 (536)	
1–3 times	35.6 (4,738)	32.6 (2,459)	39.5 (2,279)	
>3 times	49.4 (6,580)	48.6 (3,669)	50.4 (2,911)	
Do not remember/not applicable/missing	8.7 (1,159)	14.7 (1,111)	0.8 (48)	
Healthcare provider in charge of follow-up cancer care				<0.001
PCP	21.2 (2,821)	19.6 (1,478)	23.3 (1,343)	
Oncologist	41.1 (5,478)	40.5 (3,058)	41.9 (2,420)	
Both	30.5 (4,059)	30.2 (2,276)	30.9 (1,783)	
No one/unsure	7.2 (961)	9.7 (733)	3.9 (228)	
PCP involvement in follow-up cancer care				<0.001
Not at all involved	12.1 (1,606)	11.2 (844)	13.2 (762)	
Not very involved/somewhat involved	49.7 (6,626)	45.9 (3,461)	54.8 (3,165)	
Very involved	35.9 (4,777)	39.9 (3,014)	30.5 (1,763)	
Missing	2.3 (310)	3.0 (226)	1.5 (84)	
Care coordination among healthcare providers involved in follow-up cancer care				<0.001
Very good/good	62.2 (8,282)	61.5 (4,642)	63.0 (3,640)	
Fair/poor/very poor	16.9 (2,249)	14.9 (1,127)	19.4 (1,122)	
Missing	20.9 (2,788)	23.5 (1,776)	17.5 (1,012)	
Received a care plan after cancer treatment				<0.001
Yes	36.5 (4,862)	34.2 (2,584)	39.5 (2,278)	
No	38.4 (5,108)	34.4 (2,598)	43.5 (2,510)	
Not applicable/blank	25.1 (3,349)	31.3 (2,363)	17.1 (986)	
Received or saw a copy of medical records after cancer treatment				<0.001
Yes, I had access	33.3 (4,433)	30.7 (2,320)	36.6 (2,113)	
No, I did not have access	41.6 (5,547)	38.8 (2,927)	45.4 (2,620)	
Not applicable/blank	25.1 (3,339)	30.5 (2,298)	18.0 (1,041)	

**Table 2:** Emergency service utilization in the first year after cancer treatment completion organized by personal and clinical characteristics of cancer survivors included in our study population.

Characteristic	Total	None	1–3 times	>3 times	*p* value
	*N* = 5,774	*N* = 4,523	*N* = 913	*N* = 338	
Sex					0.36
Male	2,641 (45.7%)	2,074 (45.9%)	403 (44.1%)	164 (48.5%)	
Female	3,133 (54.3%)	2,449 (54.1%)	510 (55.9%)	174 (51.5%)	
Age at diagnosis					<0.001
18–44	387 (6.7%)	270 (6.0%)	78 (8.5%)	39 (11.5%)	
45–64	2,339 (40.5%)	1,800 (39.8%)	412 (45.1%)	127 (37.6%)	
≥65	3,048 (52.8%)	2,453 (54.2%)	423 (46.3%)	172 (50.9%)	
Marital status					<0.001
Single	381 (6.6%)	269 (5.9%)	77 (8.4%)	35 (10.4%)	
Married/partnered	4,406 (76.3%)	3,483 (77.0%)	684 (74.9%)	239 (70.7%)	
Divorced/separated/widowed	950 (16.5%)	748 (16.5%)	143 (15.7%)	59 (17.5%)	
Prefer not to answer	37 (0.6%)	23 (0.5%)	9 (1.0%)	5 (1.5%)	
Coping with challenges					<0.001
Very easy/easy	3,641 (63.1%)	2,974 (65.8%)	505 (55.3%)	162 (47.9%)	
Neither easy nor hard	1,727 (29.9%)	1,277 (28.2%)	314 (34.4%)	136 (40.2%)	
Hard/very hard	385 (6.7%)	260 (5.7%)	87 (9.5%)	38 (11.2%)	
Missing	21 (0.4%)	12 (0.3%)	7 (0.8%)	2 (0.6%)	
Sharing worries					0.003
Very easy/easy	2,907 (50.3%)	2,324 (51.4%)	435 (47.6%)	148 (43.8%)	
Neither easy nor hard	1,680 (29.1%)	1,316 (29.1%)	261 (28.6%)	103 (30.5%)	
Hard/very hard	1,160 (20.1%)	865 (19.1%)	212 (23.2%)	83 (24.6%)	
Missing	27 (0.5%)	18 (0.4%)	5 (0.5%)	4 (1.2%)	
Asking questions					0.004
Very easy/easy	4,312 (74.7%)	3,424 (75.7%)	655 (71.7%)	233 (68.9%)	
Neither easy nor hard	1,061 (18.4%)	799 (17.7%)	191 (20.9%)	71 (21.0%)	
Hard/very hard	381 (6.6%)	282 (6.2%)	67 (7.3%)	32 (9.5%)	
Missing	20 (0.3%)	18 (0.4%)	0 (0.0%)	2 (0.6%)	
Current insurance					0.018
Government-sponsored	1,150 (19.9%)	868 (19.2%)	193 (21.1%)	89 (26.3%)	
Private	2,921 (50.6%)	2,290 (50.6%)	478 (52.4%)	153 (45.3%)	
No insurance	797 (13.8%)	635 (14.0%)	114 (12.5%)	48 (14.2%)	
No response/other	906 (15.7%)	730 (16.1%)	128 (14.0%)	48 (14.2%)	
Born in Canada					0.002
Yes	4,897 (84.8%)	3,854 (85.2%)	757 (82.9%)	286 (84.6%)	
No	825 (14.3%)	632 (14.0%)	150 (16.4%)	43 (12.7%)	
Prefer not to answer	52 (0.9%)	37 (0.8%)	6 (0.7%)	9 (2.7%)	
Language most spoken at home					<0.001
English	4,484 (77.7%)	3,627 (80.2%)	645 (70.6%)	212 (62.7%)	
French	1,040 (18.0%)	716 (15.8%)	217 (23.8%)	107 (31.7%)	
Other	250 (4.3%)	180 (4.0%)	51 (5.6%)	19 (5.6%)	
Highest level of education					<0.001
≤High school diploma	1,816 (31.5%)	1,393 (30.8%)	290 (31.8%)	133 (39.3%)	
<University degree	2,157 (37.4%)	1,677 (37.1%)	366 (40.1%)	114 (33.7%)	
University degree	1,686 (29.2%)	1,374 (30.4%)	234 (25.6%)	78 (23.1%)	
No response	115 (2.0%)	79 (1.7%)	23 (2.5%)	13 (3.8%)	
Population density					0.084
≤10,000 people	2,019 (35.0%)	1,558 (34.4%)	336 (36.8%)	125 (37.0%)	
10,000 to 50,000 people	946 (16.4%)	737 (16.3%)	154 (16.9%)	55 (16.3%)	
>50,000 people	2,740 (47.5%)	2,180 (48.2%)	411 (45.0%)	149 (44.1%)	
No response	69 (1.2%)	48 (1.1%)	12 (1.3%)	9 (2.7%)	
Employment					<0.001
Full- or part-time	1,986 (34.4%)	1,594 (35.2%)	298 (32.6%)	94 (27.8%)	
Sick leave/disability/unemployed	316 (5.5%)	195 (4.3%)	79 (8.7%)	42 (12.4%)	
Retired	3,204 (55.5%)	2,535 (56.0%)	482 (52.8%)	187 (55.3%)	
No response/other	268 (4.6%)	199 (4.4%)	54 (5.9%)	15 (4.4%)	
Income					<0.001
<$25,000	579 (10.0%)	404 (8.9%)	115 (12.6%)	60 (17.8%)	
$25,000 to <$50,000	1,258 (21.8%)	982 (21.7%)	208 (22.8%)	68 (20.1%)	
$50,000 to <$75,000	994 (17.2%)	780 (17.2%)	147 (16.1%)	67 (19.8%)	
≥$$75,000	1,815 (31.4%)	1,495 (33.1%)	246 (26.9%)	74 (21.9%)	
Prefer not to answer	1,128 (19.5%)	862 (19.1%)	197 (21.6%)	69 (20.4%)	
Overall quality of life					<0.001
Very poor/poor/fair	809 (14.0%)	524 (11.6%)	184 (20.2%)	101 (29.9%)	
Good/very good	4,943 (85.6%)	3,984 (88.1%)	723 (79.2%)	236 (69.8%)	
Missing	22 (0.4%)	15 (0.3%)	6 (0.7%)	1 (0.3%)	
Physical health					<0.001
Very poor/poor/fair	1,261 (21.8%)	877 (19.4%)	254 (27.8%)	130 (38.5%)	
Good/very good	4,506 (78.0%)	3,644 (80.6%)	655 (71.7%)	207 (61.2%)	
Missing	7 (0.1%)	2 (0.0%)	4 (0.4%)	1 (0.3%)	
Emotional health					<0.001
Very poor/poor/fair	1,095 (19.0%)	749 (16.6%)	238 (26.1%)	108 (32.0%)	
Good/very good	4,503 (78.0%)	3,644 (80.6%)	644 (70.5%)	215 (63.6%)	
Missing	176 (3.0%)	130 (2.9%)	31 (3.4%)	15 (4.4%)	
Unmet practical concern					<0.001
No	3,623 (62.7%)	2,958 (65.4%)	507 (55.5%)	158 (46.7%)	
Yes	2,151 (37.3%)	1,565 (34.6%)	406 (44.5%)	180 (53.3%)	
Unmet emotional concern					<0.001
No	1,793 (31.1%)	1,498 (33.1%)	222 (24.3%)	73 (21.6%)	
Yes	3,981 (68.9%)	3,025 (66.9%)	691 (75.7%)	265 (78.4%)	
Unmet physical concern					<0.001
No	1,972 (34.2%)	1,611 (35.6%)	262 (28.7%)	99 (29.3%)	
Yes	3,802 (65.8%)	2,912 (64.4%)	651 (71.3%)	239 (70.7%)	
Number of comorbidities					<0.001
None	2,240 (38.8%)	1,793 (39.6%)	348 (38.1%)	99 (29.3%)	
1-2	3,091 (53.5%)	2,407 (53.2%)	486 (53.2%)	198 (58.6%)	
≥3	443 (7.7%)	323 (7.1%)	79 (8.7%)	41 (12.1%)	
Cancer type					<0.001
Breast	2,022 (35.0%)	1,637 (36.2%)	298 (32.6%)	87 (25.7%)	
Hematologic	581 (10.1%)	381 (8.4%)	133 (14.6%)	67 (19.8%)	
Colorectal	1,163 (20.1%)	816 (18.0%)	247 (27.1%)	100 (29.6%)	
Melanoma	618 (10.7%)	533 (11.8%)	65 (7.1%)	20 (5.9%)	
Prostate	1,390 (24.1%)	1,156 (25.6%)	170 (18.6%)	64 (18.9%)	
Year of cancer diagnosis					0.25
≤2012	1,521 (26.3%)	1,182 (26.1%)	235 (25.7%)	104 (30.8%)	
2013	2,754 (47.7%)	2,180 (48.2%)	428 (46.9%)	146 (43.2%)	
≥2014	1,499 (26.0%)	1,161 (25.7%)	250 (27.4%)	88 (26.0%)	
Surgery					0.007
No	1,643 (28.5%)	1,310 (29.0%)	224 (24.5%)	109 (32.2%)	
Yes	4,131 (71.5%)	3,213 (71.0%)	689 (75.5%)	229 (67.8%)	
Radiotherapy					0.070
No	2,935 (50.8%)	2,263 (50.0%)	490 (53.7%)	182 (53.8%)	
Yes	2,839 (49.2%)	2,260 (50.0%)	423 (46.3%)	156 (46.2%)	
Systemic therapy					<0.001
No	1,131 (19.6%)	964 (21.3%)	118 (12.9%)	49 (14.5%)	
Yes	4,643 (80.4%)	3,559 (78.7%)	795 (87.1%)	289 (85.5%)	
Time of last treatment					0.045
<1 year ago	512 (8.9%)	383 (8.5%)	88 (9.6%)	41 (12.1%)	
1–3 years ago	3,139 (54.4%)	2,444 (54.0%)	517 (56.6%)	178 (52.7%)	
3–5 years ago	1,552 (26.9%)	1,225 (27.1%)	236 (25.8%)	91 (26.9%)	
No treatment	571 (9.9%)	471 (10.4%)	72 (7.9%)	28 (8.3%)	
Currently receiving or received prescribed medication to prevent cancer recurrence					0.006
No	4,014 (69.5%)	3,115 (68.9%)	651 (71.3%)	248 (73.4%)	
Yes	1,675 (29.0%)	1,350 (29.8%)	245 (26.8%)	80 (23.7%)	
Unsure/blank	85 (1.5%)	58 (1.3%)	17 (1.9%)	10 (3.0%)	
Enrolled in a clinical trial					0.009
Yes	611 (10.6%)	449 (9.9%)	110 (12.0%)	52 (15.4%)	
No	4,955 (85.8%)	3,912 (86.5%)	772 (84.6%)	271 (80.2%)	
No response	208 (3.6%)	162 (3.6%)	31 (3.4%)	15 (4.4%)	
Times visit/speak in first 12 months—family doctor/practitioner					<0.001
Not at all	1,019 (17.6%)	883 (19.5%)	104 (11.4%)	32 (9.5%)	
1–3 times	2,401 (41.6%)	1,980 (43.8%)	339 (37.1%)	82 (24.3%)	
>3 times	2,072 (35.9%)	1,461 (32.3%)	412 (45.1%)	199 (58.9%)	
Do not remember/not applicable/missing	282 (4.9%)	199 (4.4%)	58 (6.4%)	25 (7.4%)	
Times visit/speak in first 12 months—oncologist/hematologist					<0.001
Not at all	536 (9.3%)	492 (10.9%)	31 (3.4%)	13 (3.8%)	
1–3 times	2,279 (39.5%)	1,960 (43.3%)	261 (28.6%)	58 (17.2%)	
>3 times	2,911 (50.4%)	2,038 (45.1%)	612 (67.0%)	261 (77.2%)	
Do not remember/not applicable/missing	48 (0.8%)	33 (0.7%)	9 (1.0%)	6 (1.8%)	
Healthcare provider in charge of follow-up cancer care					<0.001
PCP	1,343 (23.3%)	1,125 (24.9%)	164 (18.0%)	54 (16.0%)	
Oncologist	2,420 (41.9%)	1,866 (41.3%)	420 (46.0%)	134 (39.6%)	
Both	1,783 (30.9%)	1,336 (29.5%)	311 (34.1%)	136 (40.2%)	
No one/unsure	228 (3.9%)	196 (4.3%)	18 (2.0%)	14 (4.1%)	
PCP involvement in follow-up cancer care					<0.001
Not at all involved	762 (13.2%)	628 (13.9%)	99 (10.8%)	35 (10.4%)	
Not very involved/somewhat involved	3,165 (54.8%)	2,495 (55.2%)	506 (55.4%)	164 (48.5%)	
Very involved	1,763 (30.5%)	1,339 (29.6%)	291 (31.9%)	133 (39.3%)	
Missing	84 (1.5%)	61 (1.3%)	17 (1.9%)	6 (1.8%)	
Care coordination among healthcare providers involved in follow-up cancer care					0.27
Very good/good	3,640 (63.0%)	2,791 (61.7%)	624 (68.3%)	225 (66.6%)	
Fair/poor/very poor	1,122 (19.4%)	868 (19.2%)	174 (19.1%)	80 (23.7%)	
Missing	1,012 (17.5%)	864 (19.1%)	115 (12.6%)	33 (9.8%)	
Received a care plan after cancer treatment					0.013
Yes	2,278 (39.5%)	1,769 (39.1%)	390 (42.7%)	119 (35.2%)	
No	2,510 (43.5%)	1,955 (43.2%)	399 (43.7%)	156 (46.2%)	
Not applicable/blank	986 (17.1%)	799 (17.7%)	124 (13.6%)	63 (18.6%)	
Received or saw a copy of medical records after cancer treatment					<0.001
Yes, I had access	2,113 (36.6%)	1,590 (35.2%)	372 (40.7%)	151 (44.7%)	
No, I did not have access	2,620 (45.4%)	2,070 (45.8%)	406 (44.5%)	144 (42.6%)	
Not applicable/blank	1,041 (18.0%)	863 (19.1%)	135 (14.8%)	43 (12.7%)	

**Table 3:** Multivariable analysis of factors associated with increased emergency service utilization in the first year after cancer treatment completion.

Characteristic	1–3 times		>3 times	
	RRR (95% CI)	*p* value	RRR (95% CI)	*p* value
Sex				
Male	(Base)		(Base)	
Female	0.93 (0.73–1.19)	0.58	0.92 (0.64–1.32)	0.66
Age at diagnosis				
18–44	(Base)		(Base)	
44–65	0.72 (0.52–0.99)	0.04	0.44 (0.27–0.71)	0
≥65	0.49 (0.33–0.72)	0	0.35 (0.19–0.62)	0
Marital status				
Single	(Base)		(Base)	
Married/partnered	0.72 (0.53–0.99)	0.04	0.66 (0.42–1.04)	0.07
Divorced/separated/widowed	0.66 (0.46–0.94)	0.02	0.58 (0.34–0.97)	0.04
Prefer not to answer	2.22 (0.85–5.79)	0.1	2.81 (0.73–10.78)	0.13
Coping with challenges				
Very easy/easy	(Base)		(Base)	
Neither easy nor hard	1.16 (0.95–1.42)	0.15	1.07 (0.78–1.47)	0.67
Hard/very hard	1.11 (0.77–1.59)	0.58	0.79 (0.46–1.34)	0.38
Missing	2.02 (0.48–8.43)	0.33	1.05 (0.13–8.68)	0.97
Sharing worries				
Very easy/easy	(Base)		(Base)	
Neither easy nor hard	0.87 (0.71–1.07)	0.19	0.88 (0.63–1.22)	0.43
Hard/very hard	0.89 (0.69–1.14)	0.34	0.85 (0.58–1.24)	0.4
Missing	1.75 (0.34–8.94)	0.5	2.33 (0.33–16.29)	0.39
Asking questions				
Very easy/easy	(Base)		(Base)	
Neither easy nor hard	1.12 (0.88–1.41)	0.35	0.94 (0.65–1.36)	0.73
Hard/very hard	0.88 (0.61–1.27)	0.48	0.9 (0.53–1.52)	0.7
Missing	0	0.97	0.97 (0.11–8.51)	0.98
Current insurance				
Government-sponsored	(Base)		(Base)	
Private	1.02 (0.8–1.3)	0.88	0.74 (0.51–1.06)	0.1
No insurance	0.8 (0.6–1.07)	0.14	0.74 (0.48–1.13)	0.17
No response/other	0.87 (0.65–1.16)	0.33	0.59 (0.37–0.92)	0.02
Born in Canada				
Yes	(Base)		(Base)	
No	1.1 (0.85–1.42)	0.48	0.94 (0.61–1.45)	0.79
Prefer not to answer	0.71 (0.2–2.55)	0.6	4.19 (0.95–18.53)	0.06
Language most spoken at home				
English	(Base)		(Base)	
French	1.55 (1.25–1.93)	0	2.11 (1.53–2.91)	0
Other	1.16 (0.74–1.82)	0.51	1.05 (0.5–2.22)	0.9
Highest level of education				
≤High school diploma	(Base)		(Base)	
<University degree	1.07 (0.88–1.32)	0.49	0.78 (0.57–1.07)	0.13
University degree	0.96 (0.75–1.22)	0.73	0.75 (0.52–1.1)	0.15
No response	1.35 (0.72–2.55)	0.35	0.94 (0.35–2.53)	0.9
Population density				
≤10,000 people	(Base)		(Base)	
10,000 to 50,000 people	0.76 (0.59–0.97)	0.03	0.74 (0.51–1.09)	0.13
>50,000 people	0.76 (0.63–0.92)	0.01	0.77 (0.57–1.03)	0.08
No response	1.43 (0.57–3.56)	0.44	1.6 (0.43–5.95)	0.48
Employment				
Full- or part-time	(Base)		(Base)	
Sick leave/disability/unemployed	1.19 (0.84–1.68)	0.32	1.39 (0.85–2.28)	0.19
Retired	1.41 (1.11–1.78)	0	1.54 (1.05–2.25)	0.03
No response/other	1.22 (0.82–1.81)	0.32	0.71 (0.34–1.45)	0.35
Income				
≤$25,000	(Base)		(Base)	
$25,000 to <$50,000	0.72 (0.54–0.98)	0.04	0.58 (0.37–0.91)	0.02
$50,000 to <$75,000	0.63 (0.44–0.88)	0.01	1.07 (0.67–1.73)	0.77
≥$75,000	0.58 (0.41–0.82)	0	0.72 (0.43–1.21)	0.21
Prefer not to answer	0.87 (0.63–1.2)	0.38	1.02 (0.64–1.62)	0.95
Overall quality of life				
Very poor/poor/fair	(Base)		(Base)	
Good/very good	0.68 (0.51–0.92)	0.01	0.48 (0.31–0.74)	0
Missing	1.5 (0.44–5.09)	0.52	0.29 (0.03–2.78)	0.28
Physical health				
Very poor/poor/fair	(Base)		(Base)	
Good/very good	1.01 (0.78–1.31)	0.92	0.98 (0.67–1.44)	0.92
Missing	9.43 (0.95–93.02)	0.05	9.01 (0.5–161.77)	0.14
Emotional health				
Very poor/poor/fair	(Base)		(Base)	
Good/very good	0.7 (0.56–0.89)	0	0.77 (0.55–1.09)	0.14
Missing	0.87 (0.52–1.46)	0.59	1.15 (0.56–2.32)	0.71
Unmet practical concern				
No	(Base)		(Base)	
Yes	1.17 (0.97–1.41)	0.1	1.57 (1.18–2.1)	0
Unmet emotional concern				
No	(Base)		(Base)	
Yes	1.18 (0.97–1.45)	0.1	1.28 (0.93–1.76)	0.14
Unmet physical concern				
No	(Base)		(Base)	
Yes	1.03 (0.85–1.24)	0.77	1.02 (0.76–1.37)	0.89
Number of comorbidities				
None	(Base)		(Base)	
1-2	0.94 (0.79–1.13)	0.51	1.44 (1.07–1.95)	0.02
≥3	0.97 (0.69–1.35)	0.85	1.97 (1.22–3.18)	0.01
Cancer type				
Breast	0.65 (0.47–0.9)	0.01	0.47 (0.28–0.77)	0
Hematologic	0.75 (0.52–1.07)	0.11	0.69 (0.4–1.18)	0.17
Colorectal	(Base)		(Base)	
Melanoma	0.4 (0.28–0.57)	0	0.35 (0.2–0.63)	0
Prostate	0.54 (0.4–0.71)	0	0.45 (0.29–0.69)	0
Year of cancer diagnosis				
≤2012	(Base)		(Base)	
2013	0.9 (0.73–1.12)	0.35	0.77 (0.56–1.05)	0.1
≥2014	0.94 (0.72–1.21)	0.61	0.73 (0.49–1.08)	0.12
Surgery				
No	(Base)		(Base)	
Yes	1.31 (1.01–1.71)	0.04	1.27 (0.85–1.91)	0.25
Radiotherapy				
No	(Base)		(Base)	
Yes	0.84 (0.69–1.03)	0.09	0.88 (0.65–1.2)	0.42
Systemic therapy				
No	(Base)		(Base)	
Yes	1.1 (0.83–1.46)	0.52	1.06 (0.68–1.66)	0.79
Time of last treatment				
<1 year ago	(Base)		(Base)	
1–3 years ago	0.81 (0.61–1.07)	0.14	0.72 (0.48–1.1)	0.13
3–5 years ago	0.69 (0.51–0.95)	0.02	0.65 (0.41–1.03)	0.07
No treatment	0.6 (0.4–0.9)	0.01	0.64 (0.36–1.16)	0.14
Currently receiving or received prescribed medication to prevent cancer recurrence				
No	(Base)		(Base)	
Yes	0.84 (0.64–1.1)	0.21	0.85 (0.56–1.31)	0.47
Unsure/blank	1.09 (0.56–2.13)	0.8	1.43 (0.62–3.28)	0.4
Enrolled in a clinical trial				
Yes	(Base)		(Base)	
No	0.75 (0.59–0.96)	0.02	0.54 (0.38–0.76)	0
No response	0.74 (0.45–1.22)	0.24	0.54 (0.25–1.14)	0.1
Times visit/speak in first 12 months—family doctor/practitioner				
Not at all	(Base)		(Base)	
1–3 times	1.48 (1.11–1.96)	0.01	1.37 (0.84–2.24)	0.21
>3 times	2.2 (1.63–2.97)	0	3.59 (2.2–5.85)	0
N/A^†^	1.98 (1.24–3.17)	0	3.64 (1.88–7.05)	0
Times visit/speak in first 12 months—oncologist/hematologist				
Not at all	(Base)		(Base)	
1–3 times	1.32 (0.88–1.99)	0.18	0.72 (0.38–1.35)	0.3
>3 times	2.57 (1.69–3.9)	0	3.04 (1.64–5.63)	0
N/A	2.73 (1.02–7.32)	0.05	5.04 (1.59–15.96)	0.01
Healthcare provider in charge of follow-up cancer care				
PCP	(Base)		(Base)	
Oncologist	1.11 (0.84–1.46)	0.46	0.74 (0.48–1.15)	0.18
Both	0.99 (0.77–1.28)	0.96	0.81 (0.54–1.2)	0.29
No one/unsure	0.95 (0.5–1.83)	0.88	2.15 (0.97–4.79)	0.06
PCP involvement in follow-up cancer care				
Not at all involved	(Base)		(Base)	
Not very involved/somewhat involved	0.87 (0.65–1.17)	0.36	0.76 (0.48–1.21)	0.25
Very involved	0.75 (0.54–1.05)	0.09	0.75 (0.44–1.26)	0.27
Care coordination among healthcare providers involved in follow-up cancer care				
Very good/good	(Base)		(Base)	
Fair/poor/very poor	0.85 (0.69–1.06)	0.15	1.12 (0.81–1.54)	0.51
Received a care plan after cancer treatment				
Yes	(Base)		(Base)	
No	0.85 (0.71–1.03)	0.1	1.09 (0.8–1.47)	0.59
Not applicable/blank	0.83 (0.63–1.09)	0.18	1.54 (1.04–2.3)	0.03
Received or saw a copy of medical records after cancer treatment				
Yes, I had access	(Base)		(Base)	
No, I did not have access	0.84 (0.69–1.01)	0.06	0.71 (0.53–0.95)	0.02
Not applicable/blank	0.7 (0.54–0.92)	0.01	0.41 (0.26–0.63)	0

^†^N/A = do not remember/not applicable/missing.

**Table 4:** Emergency service utilization (ESU) in the first year after cancer treatment completion versus emergency service, PCP, and oncology specialist utilization in the second and third years for cancer survivors included in our study population.

	ESU first year				
	Total	None	1–3 times	>3 times	*p* value
	*N* = 5,774	*N* = 4,523	*N* = 913	*N* = 338	
ESU second year					<0.001
Not at all	4,773 (82.7%)	4,090 (90.4%)	564 (61.8%)	119 (35.2%)	
1–3 times	328 (5.7%)	61 (1.3%)	181 (19.8%)	86 (25.4%)	
>3 times	102 (1.8%)	5 (0.1%)	8 (0.9%)	89 (26.3%)	
N/A^†^	571 (9.9%)	367 (8.1%)	160 (17.5%)	44 (13.0%)	
ESU third year					<0.001
Not at all	3,687 (63.9%)	3,079 (68.1%)	469 (51.4%)	139 (41.1%)	
1–3 times	176 (3.0%)	52 (1.1%)	81 (8.9%)	43 (12.7%)	
>3 times	53 (0.9%)	7 (0.2%)	6 (0.7%)	40 (11.8%)	
N/A	1,858 (32.2%)	1,385 (30.6%)	357 (39.1%)	116 (34.3%)	
PCP second year					<0.001
Not at all	1,391 (24.1%)	1,201 (26.6%)	150 (16.4%)	40 (11.8%)	
1–3 times	2,586 (44.8%)	2,066 (45.7%)	404 (44.2%)	116 (34.3%)	
>3 times	1,323 (22.9%)	900 (19.9%)	276 (30.2%)	147 (43.5%)	
N/A	474 (8.2%)	356 (7.9%)	83 (9.1%)	35 (10.4%)	
PCP third year					<0.001
Not at all	1,282 (22.2%)	1,089 (24.1%)	151 (16.5%)	42 (12.4%)	
1–3 times	1,971 (34.1%)	1,555 (34.4%)	312 (34.2%)	104 (30.8%)	
>3 times	753 (13.0%)	516 (11.4%)	149 (16.3%)	88 (26.0%)	
N/A	1,768 (30.6%)	1,363 (30.1%)	301 (33.0%)	104 (30.8%)	
Oncologist second year					<0.001
Not at all	1,327 (23.0%)	1,178 (26.0%)	121 (13.3%)	28 (8.3%)	
1–3 times	2,585 (44.8%)	2,063 (45.6%)	402 (44.0%)	120 (35.5%)	
>3 times	1,560 (27.0%)	1,041 (23.0%)	346 (37.9%)	173 (51.2%)	
N/A	302 (5.2%)	241 (5.3%)	44 (4.8%)	17 (5.0%)	
Oncologist third year					<0.001
Not at all	1,368 (23.7%)	1,163 (25.7%)	150 (16.4%)	55 (16.3%)	
1–3 times	2,081 (36.0%)	1,622 (35.9%)	350 (38.3%)	109 (32.2%)	
>3 times	656 (11.4%)	432 (9.6%)	137 (15.0%)	87 (25.7%)	
N/A	1,669 (28.9%)	1,306 (28.9%)	276 (30.2%)	87 (25.7%)	

^†^N/A = do not remember/not applicable/missing.

## Data Availability

The data that support the findings of this study are available from the corresponding author upon reasonable request.

## Supplementary Materials

Supplementary Materials The supplementary material contains selected questions and answer options from the Transition Study survey that may be associated with cancer survivors' ESU and used in this study. It also describes the binary variable assessing unmet needs among cancer survivors created for this study based on methods previously used in the publication of the Transition Study results.
