# Supplementary material for: Variations in Emergency Service Utilization among Cancer Survivors: Results from the Pan-Canadian Experiences of Cancer Patients in Transition Study Survey
Source: J Oncol. 2023 Mar 16;2023:5056408. doi: 10.1155/2023/5056408 (PMC10036192; doi:10.1155/2023/5056408)
Supplement: Supplementary Materials — The supplementary material contains selected questions and answer options from the Transition Study survey that may be associated with cancer survivors' ESU and used in this study. It also describes the binary variable assessing unmet needs among cancer survivors created for this study based on methods previously used in the publication of the Transition Study results. [file 5056408.f1.docx]

Supplementary Material

**Table 1: Selected Transition Study survey questions and answers used in this study**

| **Survey Question Number^†^** | **Questions** | **Answer Options** |
| --- | --- | --- |
| 2 | Are you…? | - Male - Female - Other (specify): _____ - Prefer not to answer |
| 3 | How old are you? | - Under 18 - 18 to 24 - 25 to 29 - 30 to 34 - 35 to 44 - 45 to 54 - 55 to 64 - 65 to 74 - 75 to 84 - 85 or over - Prefer not to answer |
| 4 | Are you currently…? | - Single (never married) - Married - Partnered (living with someone) - Separated - Divorced - Widowed - Prefer not to answer |
| 8 | How would you describe your overall quality of life today? | - Very good - Good - Fair - Poor - Very poor |
| 9a | In general, would you say your physical health is… | - Very good - Good - Fair - Poor - Very poor |
| 9b | In general, would you say your emotional health is… | - Very good - Good - Fair - Poor - Very poor |
| 10 | Which, if any, of the following chronic conditions did you have **before** you were diagnosed with cancer? | - Arthritis, osteoarthritis, or other rheumatic disease - Cardiovascular or heart condition; hypertension or high blood pressure - Chronic kidney disease - Diabetes - Osteoporosis - Respiratory diseases (such as asthma or COPD - chronic obstructive pulmonary disease) - Mental health issues (such as depression or anxiety) - Other chronic condition. Specify type: _____ - No chronic conditions |
| 11 | Generally, how easy or hard do you find each of the following?   - Coping with challenges in your day-to-day life - Sharing your worries or concerns with others - Asking doctors questions about your concerns related to follow-up cancer care | - Very easy - Easy - Neither easy nor hard - Hard - Very hard |
| 13 | What type of cancer did you have? | - Bladder - Blood cancer/haematological (select one):   Hodgkin lymphoma  Diffuse B-cell lymphoma  Acute lymphocytic leukemia  Acute myelogenous leukemia  All other types of leukemia, non-Hodgkin lymphoma, myeloma  Not sure what type of blood cancer   - Brain/Central nervous system - Breast - Colorectal (colon or rectal cancer) - Gynaecological (cervical, ovarian, uterine, or fallopian tube) - Melanoma skin cancer (not basal cell carcinoma or squamous cell carcinoma) - Prostate - Sarcoma - Stomach or esophagus - Testicular - Thyroid - Other (specify): _____ |
| 14 | In what year were you diagnosed with this cancer? | - Prior to 2010 - 2010 - 2011 - 2012 - 2013 - 2014 - 2015 |
| 16 | What type(s) of cancer treatment did you receive for this cancer, if any? | - Surgery - Chemotherapy (intravenous or oral) - Drug therapy, such as: Immunotherapy/biologic therapy (e.g., antibodies, vaccines) or Hormone therapy (e.g., Tamoxifen, androgen deprivation) - Radiation therapy (external-beam radiation therapy or brachytherapy/internal radiation therapy) - Bone marrow or stem cell transplant - Alternative medicine (e.g., traditional medicine healer, homeopathy, acupuncture, Indigenous healing practices) - Other (specify): _____ - I have **not** received any cancer treatment and have **no plan for treatment** - I have **not** received any cancer treatment, but I am **being closely monitored in case I need treatment** |
| 17 | When was the last time you received **any** type of cancer treatment in a cancer treatment centre or hospital for this cancer? | - Within the past six months - Six months to less than 1 year ago - 1 to less than 2 years ago - 2 to less than 3 years ago - 3 to less than 5 years ago - 5 or more years ago - I have not received cancer treatment |
| 18 | Did you ever receive, or are you currently receiving, any prescribed medicines to prevent cancer from recurring or to prevent another type of cancer? | - Yes (specify): _____ - No - Unsure |
| 19 | Did you participate in a cancer clinical trial (research study) at a cancer centre, cancer clinic or hospital for this cancer? | - Yes - No - Unsure |
| 20 | Since completing your cancer treatment, which physician has been in charge of overseeing your follow-up cancer care? | - Family doctor/general practitioner/nurse practitioner - Your oncologist, hematologist, surgeon, or other cancer specialist - Both - No one - Unsure |
| 21 | How involved is your family doctor/general practitioner/nurse practitioner in your follow-up cancer care? | - Very involved - Somewhat involved - Not very involved - Not at all involved - I do not have a family doctor/general practitioner/nurse practitioner - Unsure |
| 30 | How would you describe the coordination between your various doctors and health care providers when it comes to your follow-up cancer care (i.e., do they know what each other is doing)? | - Very good - Good - Fair - Poor - Very poor - Unsure - Not applicable |
| 68 | Did you receive a formal written plan for your follow-up cancer care from the cancer centre/clinic/hospital where you received cancer treatment? | - Yes, I got a plan **before** I completed cancer treatment - Yes, I got a plan **after** I completed cancer treatment - No, I heard about a plan but never got one - No, I never heard of a plan - Not applicable |
| 69 | After completing cancer treatment, did you see or get a copy of your medical records, charts, prescription history, etc. when you wanted to? | - Yes, I had access - Yes, I had access but I wasn’t interested - Yes, I had access but I didn’t want to pay for it - No, I didn’t know I could have access - Not applicable |
| 71 | What type of health insurance coverage did you have after your cancer treatment was completed? | - A government-sponsored plan, such as Drug Benefit for those over 65, or disability benefits - My employer-sponsored benefit plan - My spouse’s or my parent’s employer-sponsored benefit plan - A plan sponsored through an association such as a union, trade association, or through my post-secondary institution - Other, such as my own private plan purchased from an insurance company - I do not have an insurance plan - Don’t know |
| 73 | Were you born in Canada? | - Yes - No - Prefer not to answer |
| 75 | What language(s) do you speak most often at home? | - English - French - An Indigenous language - Arabic - Chinese/Cantonese - Chinese/Mandarin - German - Italian - Korean - Persian (Farsi) - Polish - Portuguese - Punjabi - Russian - Spanish - Tagalog - Tamil - Urdu - Vietnamese - Other, specify: _____ - Prefer not to answer |
| 76 | What is your highest level of education? | - Grade school or less - Some high school - High school diploma or certificate - Some college or technical school/CEGEP - College or technical school/CEGEP - Some university - University undergraduate degree (Bachelor’s) - University graduate degree (Master’s or PhD) - Prefer not to answer |
| 78 | Which one of the following best describes where you currently live? | - On an acreage, ranch or farm - In a town (less than 2,000 people) - In a town (2,000 to 10,000 people) - In a small city (10,000 to 50,000 people) - In a large city (more than 50,000 people) |
| 79 | Which **one** of the following best describes your current employment situation? | - Working full-time - Working part-time - On vacation or paid leave - On paid sick leave/disability leave due to cancer - On paid sick leave/disability leave due to other reason - I am a homemaker/stay-at-home parent - I am a full-time student - I am retired - I am currently unemployed - Prefer not to answer |
| 80 | What is your total annual household income **before taxes**? | - Less than $25,000 - 25,000 to less than $50,000 - $50,000 to less than $75,000 - $75,000 to less than $125,000 - $125,000 or more - Prefer not to answer |
| **Outcome** | | |
| 23 | To the best of your memory, thinking about issues related to your follow-up cancer care, how many times did you visit or speak to each of the following, (a) in the first 12 months after you completed your treatment, (b) in the second year after you completed your treatment, and (c) in the third year after you completed your treatment?   - Family doctor/general practitioner/nurse practitioner - Your oncologist, hematologist, surgeon, or other cancer specialist - Emergency rooms or urgent care centres | - 5 or more times - 3-4 times - 1-2 times - Not at all - Don’t remember - Not applicable |

† The survey question number refers to the question number from the original survey.

***Healthcare Utilization During Follow-Up Cancer Care Variables Creation***

We created a variable assessing unmet needs among cancer survivors; a binary variable derived from methods previously used in the publication of the Transition Study results described by Shakeel et al.^17^

This variable was defined using responses to question 23 of the Transition Study survey. This question asked respondents how often they visited an emergency room or urgent care centre (herein referred to as emergency services) in the first year after primary cancer treatment. The responses were aggregated into three categories for the first year after cancer treatment (namely, not at all, one to three times, and more than three times) with aim to capture varying degrees of healthcare utilization and to help with patient anonymity by including at least five participants per category. For the second and third years, the responses were aggregated into four categories (namely, not applicable/do not remember, not at all, one to three times, and more than three times).

The secondary outcome of this study was to examine further healthcare utilization in the first three years following cancer treatment. In question 23, respondents answered the following prompt “To the best of your memory, thinking about issues related to your follow-up cancer care, how many times did you visit or speak to each of the following?”, here they were asked about the number of times they visited or spoke with their family doctor/general practitioner/nurse practitioner (herein referred to as primary care provider (PCP)) and oncologist/hematologist/surgeon/other cancer specialist (herein referred to as oncology specialist). They were asked to rate their utilization during three time intervals: (1) in the first year after cancer treatment, (2) in the second year after treatment, and (3) in the third year after treatment. Their responses could include one of the following five options: not applicable/do not remember, not at all, one to two times, three to four times, or five or more times. These response categories were aggregated into four categories: not applicable/do not remember, not at all, one to three times, and more than three times, to capture the varying degrees of healthcare utilization after cancer care completion.
